# HCC, diet and metabolic factors: Diet and HCC

**Published:** 2011-03-01

**Authors:** Maurizio Montella, Anna Crispo, Aldo Giudice

**Affiliations:** 1 Epidemiology Unit, National Cancer Institute, Napoli, Italy

**Keywords:** Hepatocellular carcinoma, Diet, Alcohol, Obesity, Diabetes mellitus, Coffee

## Abstract

Hepatocellular carcinoma is the most common primary liver malignancy and is an international public health concern, constituting one of the most deadly cancers worldwide. Infection with hepatitis B virus and hepatitis C virus is a major risk factor for HCC in developed countries. Emerging evidence indicates that there are other important lifestyle factors that contribute to the international burden of HCC, such as alcohol consumption, diabetes, obesity, and the intake of aflotoxin-contaminated food. Obesity and diabetes are also likely to be risk factors for HCC, the most frequent subtype of liver cancer. The chief pathway by which obesity increases risk involves the association between obesity and nonalcoholic fatty liver disease (NAFLD). Coffee consumption has been studied extensively and appears to have a favorable effect on the prevention of liver diseases, including HCC. One hypothesis suggests that coffee intake lowers serum levels of gamma-glutamyltransferase (GGT), which is associated with a lower incidence of HCC. It is estimated that more than 80% of HCC cases are attributable to four principal causes that are avoidable. It is difficult to make dietary recommendations, because it is unknown whether consuming higher amounts of specific antioxidants will decrease the risk of developing hepatocellular carcinoma. A diet rich that is in polyunsaturated fatty acids and, possibly, B-carotene could reduce the risk of HCC, and high dietary GL is associated with an increased risk independently of cirrhosis or diabetes.

Hepatocellular carcinoma (HCC) is the most common primary liver malignancy and represents an international public health concern, constituting one of the most deadly cancers worldwide [1]. Infection with hepatitis B virus (HBV) and hepatitis C virus (HCV) is a major risk factor for HCC in developed countries [2]. Over the past several decades, HCC incidence and mortality have increased in the United States, Japan, and several European countries [1][2][3][4][5]. Similar trends in obesity have implicated an association between the risk of HCC and excessive weight. A recent meta-analysis, which summarized the evidence from cohort studies, noted increased HCC risks of 17% for overweight people and 90% for obese persons, compared with those with normal weight[6][7]. Infection with hepatitis C (HCV) and/or hepatitis B (HBV) virus and elevated alcohol consumption are leading risk factors for HCC, accounting for ~90% of cases (BIBMM). Other factors might also contribute, including medical conditions, smoking habits, and diet [8].

Emerging evidence indicates that the etiology of many cases of HCC is multifactorial, comprising infectious origins, comorbid conditions, and environmental exposure. Accordingly, many cases are attributed to the growing prevalence of chronic infections with hepatitis B and hepatitis C viruses [1][2][3][4][5][6][7][8][9]. However, there are other important lifestyle factors that contribute to the international burden of HCC, such as alcohol consumption, diabetes, obesity, and intake of aflotoxin-contaminated food [8][10][11][12]. Alcohol consumption increases the risk of HCC primarily through the development of cirrhosis. This risk appears to be proportional to the amount of alcohol that is consumed [13]. In addition, an association between genetic polymorphisms of enzymes (e.g., aldehyde dehydrogenase 2) that mediate the metabolism of ethanol and an increased risk of HCC in heavy alcohol drinkers has been also proposed as a mechanism through which HCC develops [14].

Obesity is another risk factor for several chronic diseases, including hypertension, insulin resistance (IR), type 2 diabetes, dyslipidemia, and chronic heart disease [15]. Collectively, abdominal obesity, IR, dyslipidemia, and elevated blood pressure are known as the metabolic syndrome, reflecting overnutrition, a sedentary lifestyle, and resultant excess adiposity, which is associated with a chronic proinflammatory state [15][16]. Notably, obesity and diabetes are also likely to be risk factors for HCC, the most frequent subtype of liver cancer. The chief pathway by which obesity increases risk involves the association between obesity and nonalcoholic fatty liver disease (NAFLD) [17]. NAFLD is the most common cause of chronic liver disease among adults in western countries [18]. NAFLD comprises a spectrum of conditions, ranging from fat alone to fat plus inflammation, fat plus ballooning degeneration, and nonalcoholic steatohepatitis (NASH), which is a well-recognized cause of cirrhosis and has been increasingly associated with the development of carcinoma.

As the hepatic entity of metabolic syndrome, NAFLD/NASH is a risk factor for HCC, even in the absence of cirrhosis [19]. A recent study has shown that NAFLD is a principal risk factor in the development of HCC, irrespective of age [20]. Accumulating evidence also suggests that visceral adipose tissue secretes vascular endothelial growth factor (VEGF) and other adipokines, implicating the dysregulation of angiogenesis as a connection between obesity and worse clinical outcome [21]. In animal models, leptin promotes angiogenesis and thus can facilitate the progression of NASH to HCC [22]. Leptin also activates many signal transduction pathways, such as JNK, protein kinase B, AKT, and the extracellular signal-regulated kinase pathway in HCC cells, all of which promote the progression of cancer [23].

In addition to increasing the prevalence of chronic liver disease, diabetes is an independent risk factor for the development of HCC. In a recent systematic review of 13 case control studies, 11 reports supported an association between diabetes and the development of HCC [24]. Of the 13 case control studies, subjects with diabetes had a 2-fold higher risk of HCC [24]. The presence of diabetes remained an independent risk factor of HCC after adjusting for alcohol use or viral hepatitis [10][11]. Many case reports and case reviews of HCC in NASH have supported the association of diabetes and obesity with the risk of HCC and have implicated age and advanced fibrosis as significant risks. Insulin resistance and the resulting inflammatory cascade, which are associated with the development of NASH, appear to mediate carcinogens go HCC. The incidence of NASH is expected to increase with the growing epidemic of diabetes and obesity [25].

Coffee consumption has also been extensively studied and appears to have a favorable effect on the prevention of liver diseases, including HCC [26]. There are several hypotheses that explain why coffee attenuates the risk of HCC. One hypothesis suggests that coffee intake lowers serum levels of gamma-glutamyltransferase (GGT), which is associated with a lower incidence of HCC [26]. Coffee consumption has also been linked to a lower incidence of cirrhosis, which is a major risk factor for the development of HCC [27][28]. Several studies have examined whether alterations in diet affect the risk of HCC. For example, a trial from Italy studied a broad range of dietary habits in 185 patients with HCC and 142 patients without cancer. Those with HCC were more likely to consume more calories, were 5 times more likely to be former drinkers, and were 30 times more likely to be infected with HCV or hepatitis B virus [29]. The consumption of iron and thiamine was associated with a significant 3-fold and 2-fold increase in the risk of HCC, respectively. In contrast, a-carotene and linoleic acid consumption was associated with a reduced risk of HCC [29][30].

In a similar study, in the highest quartile of consumers of yogurt and milk, the intake of white meat and eggs correlated with a significantly lower likelihood of the development of HCC. This effect was observed in patients with and without viral hepatitis [12]. Other studies have recently shown that red meat and saturated fat are associated with increased chronic liver disease (CLD) and hepatocellular carcinoma [31]. Several mechanisms, involving fat, iron, heterocyclic amines, and N-nitroso compounds, link meat intake with chronic liver disease and hepatocellular carcinoma. Notably, amino acid-defined diets that are deficient in methyl groups effect a high incidence of HCC. A methyl-deficient diet can induce liver injuries that resemble human nonalcoholic steatohepatitis, one of the principal risk factors for the development of HCC. Such a diet disrupts DNA methylation by causing a profound loss of global cytosine methylation, predominantly at heavily methylated repetitive sequences. As a consequence, many genes are differentially expressed and correlate inversely with the extent of CpG island methylation [32]. Furthermore, these genes are associated with altered lipid and glucose metabolism, DNA damage and repair, apoptosis, the development of fibrosis, and liver tissue remodeling.

Another important risk factor for the development of HCC is the contamination of foodstuffs with aflotoxin B1 (AFB1). AFB1 is a mycotoxin that is produced by the fungus Aspergillus, which grows readily on food when it is stored under warm, damp conditions [33][34]. When ingested, it is metabolized into the active AFB1-exo-8, 9-epoxide, which binds to DNA and causes damage, such as mutations in the p53 tumor suppressor gene. This mutation has been reported in 50% of HCC tumors in southern Africa, where aflotoxin B1 is a known risk factor of HCC [33][34][35]. A prospective case control study from China of 18244 middle-aged men demonstrated that individuals who expressed urinary aflotoxin biomarkers had a significantly greater risk of HCC after adjustments for HBV surface antigen seropositivity and cigarette smoking. Recent research suggests that the intake of several dietary antioxidants (e.g., coenzyme Q[12], vitamin C and E, selenium) and phytochemicals (e.g., ellagic acid, curcumin, lycopene, epigallocatechin gallate, and resveratrol) that are present in fruit, vegetables, herbs and medicinal plants can prevent cardiovascular abnormalities, neurodegeneration, and hepatocarcinogenesis [36][37]. These phytochemicals not only have antioxidant properties but also activate cellular stress response pathways through the induction of kinases and transcription factors, leading to the expression of antioxidants and phase II enzymes. Activation of the Nrf2 transcription factor-antioxidant response element (ARE) pathway by these phytochemicals effects cytoprotection and chemoprevention [38].

However, the hermetic effects of these phytochemicals must be considered, because at low doses they have stimulatory effects but are toxic at higher doses [39]. Various studies from Japan and Europe have found that those who consume large amounts of green vegetables have a significantly lower risk of developing HCC [39][40]. One study has demonstrated that the daily consumption of green vegetables, compared with several times per week, has a protective effect against the development of HCC. In contrast, a Greek study failed to note an association between vegetable intake and a reduction in the risk of developing HCC [41]. In conclusion, more than 80% of HCC cases are estimated to be attributed to four principal causes that are avoidable. Hepatitis-negative HCC in men is effected primarily by heavy alcohol consumption. Diet is involved in the risk of HCC; specifically, the beneficial effect of certain dietary products, such as fruits and vegetables, is independent of other major risk factors (e.g., HBV and HCV infections).

Due to conflicting results, it is difficult to make dietary recommendations, because it is unknown whether consuming higher amounts of specific antioxidants will decrease the risk of developing hepatocellular carcinoma. A diet that is rich in polyunsaturated fatty acids and, possibly, B-carotene can reduce the risk of HCC, and high dietary GL is associated with increased risk, independently of cirrhosis or diabetes. Coffee has favorable effects, as shown in many studies, especially in HCV infection.

## References

[1] Llovet JM, Burroughs A, Bruix J (2003). Hepatocellular carcinoma. Lancet.

[2] Yu MC, Yuan JM, Govindarajan S, Ross RK (2000). Epidemiology of hepatocellular carcinoma. Can J Gastroenterol.

[3] El-Serag HB (2004). Hepatocellular carcinoma: recent trends in the United States. Gastroenterology.

[4] La Vecchia C, Lucchini F, Franceschi S, Negri E, Levi F (2000). Trends in mortality from primary liver cancer in Europe. Eur J Cancer.

[5] Shibuya K, Yano E (2005). Regression analysis of trends in mortality from hepatocellular carcinoma in Japan, 1972-2001. Int J Epidemiol.

[6] Blonski W, Kotlyar DS, Forde KA (2010). Non-viral causes of hepatocellular carcinoma. World J Gastroenterol.

[7] Clark JM (2006). The epidemiology of nonalcoholic fatty liver disease in adults. J Clin Gastroenterol.

[8] Franceschi S, Montella M, Polesel J, La Vecchia C, Crispo A, Dal Maso L, Casarin P, Izzo F, Tommasi LG, Chemin I, Trépo C, Crovatto M, Talamini R (2006). Hepatitis viruses, alcohol, and tobacco in the etiology of hepatocellular carcinoma in Italy. Cancer Epidemiol Biomarkers Prev.

[9] Montella M, Crispo A, Izzo F, Ronga D, Tamburini M, De Marco M, Tridente V, Desicato S, Fabbrocini G, Cuomo O (2000). HCV and hepatocellular carcinoma: A case-control study in an area of hyperendemicity. Int J Mol Med.

[10] Polesel J, Zucchetto A, Montella M, Dal Maso L, Crispo A, La Vecchia C, Serraino D, Franceschi S, Talamini R (2009). The impact of obesity and diabetes mellitus on the risk of hepatocellular carcinoma. Ann Oncol.

[11] Rossi M, Lipworth L, Maso LD, Talamini R, Montella M, Polesel J, McLaughlin JK, Parpinel M, Franceschi S, Lagiou P, La Vecchia C (2009). Dietary glycemic load and hepatocellular carcinoma with or without chronic hepatitis infection. Ann Oncol.

[12] Talamini R, Polesel J, Montella M, Dal Maso L, Crispo A, Tommasi LG, Izzo F, Crovatto M, La Vecchia C, Franceschi S (2006). Food groups and risk of hepatocellular carcinoma: A multicenter case-control study in Italy. Int J Cancer.

[13] Donato F, Tagger A, Gelatti U, Parrinello G, Boffetta P, Albertini A, Decarli A, Trevisi P, Ribero ML, Martelli C, Porru S, Nardi G (2002). Alcohol and hepatocellular carcinoma: the effect of lifetime intake and hepatitis virus infections in men and women. Am J Epidemiol.

[14] Munaka M, Kohshi K, Kawamoto T, Takasawa S, Nagata N, Itoh H, Oda S, Katoh T (2003). Genetic polymorphisms of tobacco- and alcohol-related metabolizing enzymes and the risk of hepatocellular carcinoma. J Cancer Res Clin Oncol.

[15] Semenkovich CF (2006). Insulin resistance and atherosclerosis. J Clin Invest.

[16] Siegel AB, Zhu AX (2009). Metabolic syndrome and hepatocellular carcinoma: two growing epidemics with a potential link. Cancer.

[17] Sanyal AJ, Yoon SK, Lencioni R (2010). The etiology of hepatocellular carcinoma and consequences for treatment. Oncologist.

[18] Ascha MS, Hanouneh IA, Lopez R, Tamimi TA, Feldstein AF, Zein NN (2010). The incidence and risk factors of hepatocellular carcinoma in patients with nonalcoholic steatohepatitis. Hepatology.

[19] Ertle J, Dechêne A, Sowa JP, Penndorf V, Herzer K, Kaiser G, Schlaak JF, Gerken G, Syn WK, Canbay A (2010). Nonalcoholic fatty liver disease progresses to HCC in the absence of apparent cirrhosis. Int J Cancer.

[20] Cheung O, Sanyal AJ (2009). Recent advances in nonalcoholic fatty liver disease. Curr Opin Gastroenterol.

[21] Rega G, Kaun C, Demyanets S, Pfaffenberger S, Rychli K, Hohensinner PJ, Kastl SP, Speidl WS, Weiss TW, Breuss JM, Furnkranz A, Uhrin P, Zaujec J, Zilberfarb V, Frey M, Roehle R, Maurer G, Huber K, Wojta J (2007). Vascular endothelial growth factor is induced by the inflammatory cytokines interleukin-6 and oncostatin m in human adipose tissue in vitro and in murine adipose tissue in vivo. Arterioscler Thromb Vasc Biol.

[22] Ikejima K, Takei Y, Honda H, Hirose M, Yoshikawa M, Zhang YJ, Lang T, Fukuda T, Yamashina S, Kitamura T, Sato N (2002). Leptin receptor-mediated signaling regulates hepatic fibrogenesis and remodeling of extracellular matrix in the rat. Gastroenterology.

[23] Saxena NK, Sharma D, Ding X, Lin S, Marra F, Merlin D, Anania FA (2007). Concomitant activation of the JAK/STAT, PI3K/AKT, and ERK signaling is involved in leptin-mediated promotion of invasion and migration of hepatocellular carcinoma cells. Cancer Res.

[24] El-Serag HB, Hampel H, Javadi F (2006). The association between diabetes and hepatocellular carcinoma: a systematic review of epidemiologic evidence. Clin Gastroenterol Hepatol.

[25] Tokushige K, Hashimoto E, Yatsuji S, Tobari M, Taniai M, Torii N, Shiratori K (2010). Prospective study of hepatocellular carcinoma in nonalcoholic steatohepatitis in comparison with hepatocellular carcinoma caused by chronic hepatitis C. J Gastroenterol.

[26] Gallus S, Bertuzzi M, Tavani A, Bosetti C, Negri E, La Vecchia C, Lagiou P, Trichopoulos D (2002). Does coffee protect against hepatocellular carcinoma?. Br J Cancer.

[27] Montella M, Polesel J, La Vecchia C, Dal Maso L, Crispo A, Crovatto M, Casarin P, Izzo F, Tommasi LG, Talamini R, Franceschi S (2007). Coffee and tea consumption and risk of hepatocellular carcinoma in Italy. Int J Cancer.

[28] Tanaka K, Tokunaga S, Kono S, Tokudome S, Akamatsu T, Moriyama T, Zakouji H (1998). Coffee consumption and decreased serum gamma-glutamyltransferase and aminotransferase activities among male alcohol drinkers. Int J Epidemiol.

[29] Polesel J, Talamini R, Montella M, Maso LD, Crovatto M, Parpinel M, Izzo F, Tommasi LG, Serraino D, La Vecchia C, Franceschi S (2007). Nutrients intake and the risk of hepatocellular carcinoma in Italy. Eur J Cancer.

[30] Yuan JM, Gao YT, Ong CN, Ross RK, Yu MC (2006). Prediagnostic level of serum retinol in relation to reduced risk of hepatocellular carcinoma. J Natl Cancer Inst.

[31] Freedman ND, Cross AJ, McGlynn KA, Abnet CC, Park Y, Hollenbeck AR, Schatzkin A, Everhart JE, Sinha R (2010). Association of meat and fat intake with liver disease and hepatocellular carcinoma in the NIH-AARP cohort. J Natl Cancer Inst.

[32] Tryndyak VP, Han T, Muskhelishvili L, Fuscoe JC, Ross SA, Beland FA, Pogribny IP (2010). Coupling global methylation and gene expression profiles reveal key pathophysiological events in liver injury induced by a methyl-deficient diet. Mol Nutr Food Res.

[33] Bressac B, Kew M, Wands J, Ozturk M (1991). Selective G to T mutations of p53 gene in hepatocellular carcinoma from southern Africa. Nature.

[34] El-Serag HB (2007). Translational research: the study of community effectiveness in digestive and liver disorders. Gastroenterology.

[35] Glauert HP, Calfee-Mason K, Stemm DN, Tharappel JC, Spear BT (2010). Dietary antioxidants in the prevention of hepatocarcinogenesis: a review. Mol Nutr Food Res.

[36] Johnson LJ, Meacham SL, Kruskall LJ (2003). The antioxidants--vitamin C,vitamin E, selenium, and carotenoids. J Agromedicine.

[37] Kwak MK, Itoh K, Yamamoto M, Kensler TW (2002). Enhanced expression of the transcription factor Nrf2 by cancer chemopreventive agents: role of antioxidant response element-like sequences in the nrf2 promoter. Mol Cell Biol.

[38] Son TG, Camandola S, Mattson MP (2008). Hormetic dietary phytochemicals. Neuromolecular Med.

[39] Sauvaget C, Nagano J, Hayashi M, Spencer E, Shimizu Y, Allen N (2003). Vegetables and fruit intake and cancer mortality in the Hiroshima/Nagasaki Life Span Study. Br J Cancer.

[40] Yu MW, Hsieh HH, Pan WH, Yang CS, CJ CH (1995). Vegetable consumption, serum retinol level, and risk of hepatocellular carcinoma. Cancer Res.

[41] Kuper H, Tzonou A, Lagiou P, Mucci LA, Trichopoulos D, Stuver SO, Trichopoulou A (2000). Diet and hepatocellular carcinoma: a case-control study in Greece. Nutr Cancer.

